# Iodine Excretion and Intake in Women of Reproductive Age in South Australia Eating Plant-Based and Omnivore Diets: A Pilot Study

**DOI:** 10.3390/ijerph18073547

**Published:** 2021-03-29

**Authors:** Jane S. Whitbread, Karen J. Murphy, Peter M. Clifton, Jennifer B. Keogh

**Affiliations:** Clinical and Health Sciences, University of South Australia, Alliance for Research in Exercise Nutrition and Activity (ARENA), Adelaide 5001, Australia; jane.whitbread@mymail.unisa.edu.au (J.S.W.); karen.murphy@unisa.edu.au (K.J.M.); Peter.Clifton@unisa.edu.au (P.M.C.)

**Keywords:** urinary iodine concentration, dietary iodine, plant-based diet

## Abstract

Women consuming a strictly vegan/plant-based diet may be at increased risk of low iodine intake due to avoidance of animal products containing iodine. The aim of this pilot study was to determine the iodine excretion and intake in women consuming vegan/plant based diets compared with women consuming omnivore diets. Fifty-seven women (*n* = 31 plant-based, *n* = 26 omnivores), provided two spot urine samples to assess urinary iodine concentration (UIC). Two days of dietary intake were also recorded by participants. As the data were not normally distributed results are reported as median (IQR). UIC was significantly different between groups, 44 (26–66) µg/L in the vegan/plant-based group versus 64 (40–88) µg/L in omnivores (*p* < 0.05). UIC did not meet the >100 µg/L level recommended by the World Health Organization. Iodine intake was also significantly different, 78 (62–91) µg/day in the vegan/plant-based group and 125 (86–175) µg/day in the omnivores (*p* = 0.000). Iodine intake and bread intake were correlated with iodine excretion (CC 0.410–4.11, *p* = 0.003). These data indicate iodine insufficiency in both groups of women as the median values were below the minimum WHO recommendation. A larger study assessing iodine excretion in the Australian women of reproductive age who are not pregnant or breastfeeding is needed to confirm these findings.

## 1. Introduction

Iodine is an essential mineral where adequate dietary intake is required from conception and throughout life for optimal development, including intellectual development and thyroid functioning. Dietary sources of iodine include seaweed and other seafoods, fortified bread, iodized salt, eggs and dairy foods. Research indicates that adequate iodine intake before conception is necessary to ensure optimal maternal thyroid function during pregnancy, which is required for fetal intellectual development [1,2,3]. However, most pregnant women do not supplement with iodine prior to pregnancy [1]. Vegan and plant-based diets are becoming increasingly popular in Australia with nearly 12.1% of the population eating a vegetarian diet [4]. The potential health benefits of a plant-based diet include prevention and/or improved management of diabetes, obesity, heart disease, rheumatoid arthritis and metabolic syndrome [5,6,7,8,9,10]. Women consuming a strictly vegan/plant-based diet may be at increased risk of low iodine intake due to avoidance of iodine containing animal products [11]. Dairy foods have been found to be a variable source of iodine, yet are estimated to provide a significant contribution of iodine to the diet [12,13,14]. Milk alternatives are low in iodine and nearly all are not fortified with iodine in Australia. Concerns about poor iodine status in Australia prompted the mandatory fortification of non-organic bread with iodised salt in 2009 [12]. This was to improve iodine status in the population particularly with the intention of reducing the incidence of iodine-deficiency health problems, including impaired neurological conditions in children by correcting iodine deficiency in young children, females of child-bearing age and breast-feeding females [12]. Since bread fortification it was reported that people who consume 100g of iodine fortified bread per day had a 5 times the increased chance of meeting their iodine intake compared to lower bread consumption in women [15]. However women aged 14–50 years had a median intake of 60 g/day and only 8.6% of women of child-bearing age reported consuming ≥ 100 g bread per day [15]. In Australia a daily supplement of 150 µg/day of iodine is recommended for women when planning pregnancy [16]. Several studies have reported that women who consume iodine in the form of supplements or iodised salt prior to pregnancy are more likely to achieve adequate thyroid status during pregnancy [1,2,3]. Iodine status of pregnant and postpartum women in South Australia has recently been documented [17,18]. However these studies did not assess UIC prior to pregnancy. There are few Australian data on the iodine status of women choosing a vegan/plant-based diet and those who are not pregnant or breastfeeding. Therefore, the primary aim of this study was to determine the urinary iodine concentration of a group of women of reproductive age choosing a plant-based/vegan diet compared with a group of women choosing an omnivore diet in South Australia. The secondary aims were to determine the dietary intake and food sources of iodine in these women.

## 2. Materials and Methods

In a cross-sectional pilot study, women aged between 18–50 years, consuming either a vegan/plant based (*n* = 31) or omnivore diet (*n* = 26) were recruited from the general population between November 2019 and March 2020. They were recruited using social media advertisements, flyers distributed at the University of South Australia, and via the University of South Australia online participant portal. Participants self-reported consuming either vegan/plant-based diets or omnivore diets by reporting the level of compliance to a plant-based diet or how regularly they consumed animal products. Participants who usually took iodine supplements were not included in the study.

Participants attended the clinical trials facility at the University of South Australia on two occasions, one week apart. Anthropometric data and demographic details such as income and level of education were collected. Information about intake of specific sources of iodine, iodised salt, bread, dairy and seaweed as well as usual supplement intake were also collected. 

A spot urine sample was obtained at both visits. Time of day was similar for each participant but varied between participant depending on their availability. Urine samples were collected in 70 mL polypropylene containers and kept at room temperature. Iodine analysis was conducted by a National Association of Testing Authorities (NATA) accredited laboratory. Inductively coupled plasma/mass spectrometry (ICP-MS) was used to determine the concentration of iodine. The coefficient of variation for ICP-MS is 5.2 at a UIC level of 20 µg/L and 3.5 at a level of 200 µg/L. A study by May et al. (1997) found that iodine concentration determines by ICP-MS had a lower limit of detection for the assay (2 μg/L) when compared to 5 other techniques [19]. UIC results from the two urine samples two weeks apart were averaged.

Two, 24 h diet histories were collected from each participant using the smart phone application Research Food Diary (RFD) (Xyris software Australia, Version 6.0.0 202 AF 96 AB 300, 2019). The RFD method has been validated by Ambrosini (2018) who reported comparable nutrient analysis (r = 0.52–0.79) to 24-h recalls at a group level [20]. This method was chosen over 24-h recalls administered by the researcher as participants recorded their total food intake prospectively on two different days and did not rely on the participant’s memory. Each diet record was checked by the researcher with the participant. The diet records were analysed using the software FoodWorks Dietary Analysis package (Xyris Software Australia Version 10, Brisbane, Australia). Some foods in the dietary database used did not have micronutrient information, therefore nutrient information was obtained for these foods from the manufacturers and manually added or an equivalent food from a different brand was used which had available micronutrient information. When nutrients for manufactured products were not available, recipes were also manually added which closely matched the ingredients listed on the packaging and nutrient ratio on the nutrition panel. When nutrients for raw products were not available on Australian Food composition database, data from the US Department of Agriculture (USDA) Food Data Central was used. Dietary under-reporting was assessed using the Walter-Willett cut off method that excludes participants who had a dietary intake of less than 500kcal per day [21]. Using this method all food diaries were included. Mean or Median daily nutrient intake was compared to Australian NRV’s (Nutrient Reference Values) [16]. 

### 2.1. Power

A sample size of 25 participants per group was needed to detect a minimum significant difference in UIC of 40 µg/L at 80% power between the plant based/vegan and omnivore groups at *p* < 0.05. 

### 2.2. Statistics

Data analyses were performed using IBM SPSS statistics version 26, 2019 (Armonk, New York, NY, USA). Normality was assessed using the Q-Q test and histograms, where *p* < 0.05 was considered not normal in the Shapiro-Wilk test. Non-normally distributed data is presented as a median with IQR (25–75) and normally distributed data is presented as a mean ± SD. Categorical data was presented as a number and compared between groups using Chi square tests. Mann Whitney U tests were used to compare continuous nonparametric data between groups and paired t-tests were used to compare parametric continuous data between groups. Spearman’s test was used to assess correlations between continuous data and UIC. Independent samples Kruskal Wallis tests were used to compare differences in UIC between participants with specific food consumption from various categories. *p* < 0.05 was considered significantly different, unless otherwise specified. 

The trial was registered with the Australian New Zealand Clinical Trials Registry (ACTRN 12619001546145) and Ethics approval was obtained from the University of South Australia Human Research Ethics Committee (Application No: 202118).

## 3. Results

**Figure 1:**
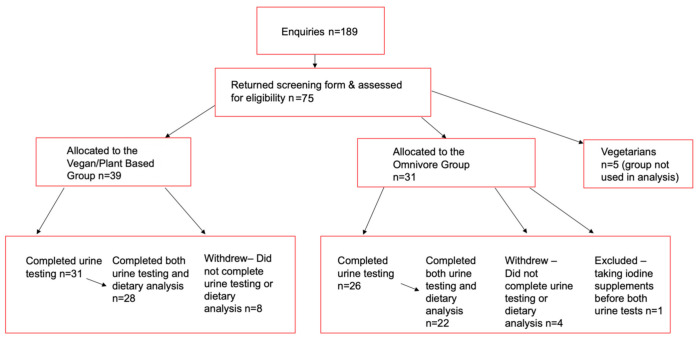
Consort diagram for vegan/plant-based, omnivore and vegetarian participants.

Participant recruitment and completion is shown in Figure 1. Of the 75 individuals that were screened for participation, *n* = 39, were identified as vegan/plant-based and *n* = 31 as omnivores. In the vegan/plant-based group 31 participants completed the urine collection and 28 participants completed both the urine analysis and diet intake records. In the omnivore group 26 participants completed the urine collection and 22 participants completed both the urine analysis and diet intake records. 

Baseline characteristics of participants who completed the urine collections are presented in Table 1. No significant difference in age, BMI, weight, level of education or income were found between the groups. 

### 3.1. Urinary Iodine Concentration

Urinary iodine concentration is shown in Table 2. Neither group of participants met the WHO guideline for a UIC >100 µg/L. Median UIC was significantly lower in vegans than omnivores (*p* = 0.044).

### 3.2. Food Sources of Iodine

Iodine containing food, including iodised salt, bread, milk, dairy, egg, fish and seaweed, intake is shown in Table 3. Iodised bread was the main iodised food source in the Vegan/Plant Based participants and bread, dairy foods and eggs were the main source in the omnivore group. Spearman’s correlation coefficient (CC) of 0.410 shows a positive linear association between iodine intake and iodine excretion (*p* = 0.003). Grams of bread intake recorded by participants was significantly correlated with UIC (CC 0.411, *p* = 0.003). In the omnivore group, median total dairy intake was 1.3 (0.7–1.8) serves per day and median dairy milk consumption was 135 (81–324) ml per day, dairy sources were not correlated with iodine excretion. Egg intake was not significantly correlated with UIC (CC = 0.109, *p* = 0.451), or fish intake (CC −0.010, *p* = 0.945). In the omnivore group, median egg consumption was 5 (0–38) g per day and fish consumption was 0 (0–24) g per day. Six vegan/plant-based participants reported using seaweed in their meal plan. The only seaweed consumed was nori at an average of 2.66g a day by 6 participants and was not significantly correlated with UIC. Median iodised bread intake was not significantly different between groups. 

Macronutrient intake is shown in Table 4. Total fat, carbohydrate and sugar intakes were not significantly different between the groups. Median protein, saturated fat, trans fat, cholesterol and very long chain omega 3 (VLC N3) fats were significantly higher in the omnivore group (*p* < 0.001). Very small amounts of cholesterol were recorded in the vegan/plant-based group as some participants recorded consuming very small amounts of animal products, mainly within processed foods (e.g., cake or muffins). The vegan group had a significantly higher intake of fibre and alpha linolenic acid (ALA) (*p* < 0.048).

### 3.3. Micronutrient Intake 

Micronutrient intake is shown in Table 5. The omnivore group had a significantly higher intake of iodine *p* = 0.000. Both vegan/plant-based and omnivore groups recorded a median dietary intake less than the RDI for iodine, iron and calcium. The vegan/plant-based group had less than the estimated average requirement (EAR) for intakes of iodine, selenium, calcium and B12, and the omnivore group had less than the EAR for calcium. The vegan/plant-based group recorded lower dietary riboflavin, selenium and B12 than the RDI. The omnivore group also recorded lower thiamine and riboflavin than the RDI. Dietary selenium, zinc, sodium and B12 was higher in the omnivore group (*p* < 0.05). The vegan/plant-based group had a higher dietary intake of iron, magnesium, vitamin C, total folate (food folate + folic acid) and food folate (*p* < 0.038).

### 3.4. Iodised & Non Iodised Salt Use

Overall participants who had iodised salt at home had a median UIC of 65 (38–87) µg/L, *n* = 19 compared to participants who did not have iodised salt at home with a median UIC of 44 (26–73) µg/L, *n* = 37, but the UIC difference between iodised salt and the non iodised salt group was not significantly different (*p* = 0.146, *n* = 56). Plant based/vegan participants were not more likely to have iodised salt at home than omnivores (*p* = 0.920). Ten (33%) plant based/vegan participants had iodised salt at home and nine (35%) omnivores had iodised salt at home. Four participants from each group recorded using iodised salt daily or multiple times a day (14–16%). No significant difference was found between dietary groups in how regularly iodised salt was used at home (*p* = 0.971). Twelve participants reported using Himalayan (non-iodised) salt (*n* = 12). They had a significantly lower median UIC of 23 (18–29) µg/L compared to participants who did not record using Himalayan salt 62 (43–87) µg/L (*n* = 44) (*p* < 0.0001). Plant based/vegan participants were not more likely to use Himalayan salt than omnivore participants (*p* = 0.755). 

## 4. Discussion

The main finding of this study was that overall the participants did not meet the WHO recommendations for a UIC greater than 100 µg/L. Further, women eating a vegan/plant-based diet had a significantly lower UIC than women consuming an omnivore diet.

### 4.1. Urinary Iodine Concentration

UIC is highly variable depending on recent dietary iodine intake [22,23,24,25]. To increase reliability two samples were provided by each participant over two non-consecutive days and the average UIC was used for analysis. The World Health Organization recommends using the median UIC from spot urine samples to adequately describe the iodine status of a population [26]. Perrine et al. (2014) (*n* = 400) demonstrated that spot iodine concentration does not vary with the time of day it was taken [27]. Charlton et al. (2014) observed no additional benefit when collecting 3 versus 2 spot urine samples from participants [28]. The median UIC of 44 µg/L in the vegan/plant-based group in this study was a similar to that of a study of Norwegian vegans, but lower than results from studies in America and Slovakia [11,29,30]. The higher result in the American vegan group is likely due to the study design that includes participants that take iodine supplements. In the Slovakian study the higher UIC may be due to the mandatory iodisation of all table salt and salt used in processed foods in Slovakia [31]. The median low UIC of 64 µg/L in this group of omnivores was similar to a New Zealand study that found 55 women between the ages of 25–44 to have a median UIC of 60 (42, 86) µg/L [32]. New Zealand has similar public health measures as Australia with mandatory bread fortification with iodised salt and non-mandatory use of iodised table salt. The between-group difference of 20 µg/L was less than seen in international studies that measured the differences in median UIC between groups of vegan/plant-based groups, omnivores and vegetarians [11,29,30]. These studies had a between group difference range of 50–139 µg/L [11,29,30]. This may be partly explained by the higher intake of seafoods in Norway and eggs in Slovakia compared to the omnivores in this study. In addition the mean BMI of Slovakian and Norwegian omnivore adults was higher than in our sample, which may indicate a greater food intake by the Norwegian and Slovakian participants. 

### 4.2. Dietary Iodine Intake

Iodine intake was 78% of the EAR and 52% of the RDI in our group of vegan/plant-based women. This is similar to the iodine intakes in vegan participants in studies conducted in Denmark and England [33,34]. Estimated median iodine intake of 78 (62–91) µg in this vegan/plant-based group was higher than the English study that found mean iodine intake in their vegan group to be 24 (12.7) µg [33] but similar to the Danish study which had a median iodine intake of 65 (54–86) µg. The higher intake found in the Australian and Danish vegan/plant-based women is likely to be due to the influence of the mandatory fortification of breads with iodised salt in Australia and Denmark [22]. The median iodine intake of 125 (86–175) µg found in the omnivore diet in the present study was less than a mean iodine intake 155 µg found in a large 2016 Australian study in women (*n* = 3496) aged 14–50 [15]. These differences may be in part due to participants in the present study having a low level of bread intake. 

### 4.3. Dietary Habits

Participants in both groups had a low intake of iodised salt at home. Bread intake was strongly correlated with UIC even though both groups had a median intake of only one piece of iodised bread per day (28–36 g). One piece of bread per day is approximately half the median bread intake found in a 2016 study of bread intake in Australian women aged 14–50 [15]. Dairy foods are thought to be a rich source of iodine based on the iodine concentration reported in the Australian Food Composition Database [35]. There was no correlation between dairy foods and UIC in the omnivore group even though 95% of omnivore participants included dairy foods with the median dairy intake at approximately 1.5 serves/day. FSANZ reports that the high level of variability of iodine in dairy foods in Australia is due to geographic location of farms, supplementary feeding practices of cattle as well as the continuing use of iodophors in some sanitizing agents [16]. There are differences in iodine concentration in soils due to both the lands proximity to the ocean and the soils ability to retain iodine [36,37]. Supplementary cattle feeds may be used when there is reduced access to pastures and international studies indicate that varying types of supplemental feeds influences iodine concentration of milk [38]. Recent advances in the development of farming seaweed for cattle-feed presents an opportunity for enrichment of Australia’s milk supply with iodine. The vegan/plant-based group used similar levels of plant milks when compared to dairy milk intake in the omnivore group. There has been an increase in the use of plant derived milk intake in Australia with an average annualised almond and soy milk industry growth of 7.2% between 2015–2020 [39]. The average plant milk intake in our vegan/plant-based sample was 170mL/day. In Australia most plant milks are poor sources of iodine as they have not been fortified with iodine. One soymilk does contain seaweed, but the concentration of iodine is not publicly available. The average level of iodine in 170mL soymilk or almond milk is 0.4–2.3 µg, compared to the same amount of dairy milk which contains 39.1 µg iodine [35]. Based on this amount of plant milk intake, if plant milks were fortified with iodine to the average level of dairy milk, consumers of plant milk would increase their daily iodine intake by approximately 37 µg day. This would bring the average estimated daily intake to 115 µg/day in the vegan/plant-based group which would meet the EAR for iodine which is set at 100 µg per day. Approximately one quarter of participants in the present study reported using Himalayan salt, which is not iodised. Participants who reported using Himalayan salt, also had a low median UIC at 22 (18–29) µg/L. Further, the Himalayan salt users did not have a significantly different level of bread and dairy intake than non-Himalayan salt users and those who used Himalayan salt were not more likely to be vegan. This study has highlighted a potential problem with the increasing use of Himalayan salt and other non-iodised salt varieties in Australia.

### 4.4. Macronutrients

Participants in the vegan/plant-based group consumed less protein than omnivores, with approximately 25% of the group not meeting the RDI for protein compared to 8% of omnivores. The median protein intake of 59 (51–69) g/day was similar to a group of vegan/plant-based participants from Denmark 59 (51–57) g/day, as well as the mean protein intake in English vegans of 61.6 (±14.1) g/day [33,34]. Mean protein intake in Finnish vegans was higher at 74 ± 30 g/day [40]. The vegan/plant based participants in the present study had significantly less saturated fat, % energy from saturated fat, trans fat and cholesterol as well as more fibre than the omnivore participants, which was similar to international studies that compared one or more of these nutrients to non-vegan participants [33,34,40].

Recommended daily intakes for VLC-N3 are not currently determined instead the adequate intake (AI) is used which is based on the median Australian intake. The plant-based/vegan group had lower intakes than both the omnivore group and the AI. This is not unexpected as VLC-N3 is found mainly in seafoods and eggs. However, the plant/based group had a significantly higher intake and exceeded the AI for ALA, some of which is converted to the VLC-N3 fats eicosapentaenoic fatty acid (EPA) and docosahexaenoic fatty acid (DHA). A higher intake of ALA was also found in Finnish vegan/plant-based participants when compared to non-vegetarians [40]. In the present study three vegan/plant-based participants (10%) and three omnivores (12%) were supplementing with VLC-N3. Algal long chain omega 3 oil is available to use as a source of VLC-N3 for vegans.

### 4.5. Other Micronutrient Intakes

Selenium intake met 76% of the EAR in the vegan/plant-based group which was also similar to the Danish and English vegan studies [33,34]. Selenium is concentrated in animal meats and found in higher levels in food grown in selenium rich soils. In Finland the introduction of selenium rich fertiliser used in farming resulted in the Finnish vegans and omnivores meeting the recommended intakes of this nutrient [40]. The vegan/plant-based group had significantly higher levels of iron intake than the omnivore group. This is also reflected in the Danish, Finnish and English dietary intake studies [33,34,40]. This may be due to the increased consumption of soy products such as tofu and soymilk by vegan/plant-based participants which are sources of iron, as well as increased wholemeal foods, legumes and green leafy vegetables. Both groups met the EAR for iron, while neither group achieved RDI levels even though iron intake was higher in the vegan/plant-based group. The Nutrient Reference Values for Australia and New Zealand have recommended that vegetarians consume 80% more iron than the RDI of 18mg due to reduced bioavailability of non-heme iron [16]. This approach has not been adopted in the UK and a review by Saunders (2013) has discussed the questionable validity of using the single 1991 study to base the recommendation for vegetarians due to the unsuitability of the study design [41,42]. In the present study, one third of vegans/plant-based participants reported taking iron supplements either within a multivitamin or separately, this was not used in the dietary analysis. Mean calcium intake was less than the EAR and RDI for both vegan/plant based and omnivore groups; vegans/plant-based participants consumed 658 (±258) mg calcium (78% EAR) (66% RDI), and omnivores consumed 772 (±331) mg (92% EAR) (77% RDI). However, the mean calcium intake for both groups was slightly higher than the average of 615mg calcium intake (62% of RDI) in Australian women between 20–54 years found in a recent osteoporosis study in Victoria [43]. The vegan/plant-based group had higher levels of magnesium, beta carotene, vitamin E, vitamin C and folate, indicative of a diet higher in plant foods. A Danish study also found significantly higher levels of these nutrients in vegans [34]. Both groups did not meet the RDI for riboflavin while they did exceed the EAR. Three international studies also found that vegans had significantly lower intakes of riboflavin intake than non-vegans likely due to excluding dairy foods which are a source of this nutrient [33,34,40]. B12 intake, without supplements, was low in the vegan/plant-based group which is replicated in international studies [33,34,40]. Sixty-four percent of vegan/plant-based participants reported supplementing with B12.

### 4.6. Limitations

The small sample size in this pilot study limits the generalisability of the data collected. More than 60% of the female adult Australian population have a BMI > 25 kg/m^2^ [44], however in the present study, both groups had a median BMI between 21–22 kg/m^2^. BMI was not significantly different between groups. In our sample 60% of participants had a Bachelor degree or higher which is higher than the general adult population where 24% of people have this qualification [45]. This could be due in part that some individuals were recruited from within the University in response to internal advertisements and are not representative of the general population. Measurements of thyroid function were not assessed which meant the impact of low iodine intake on women’s thyroid function is not known. A larger sample size would increase accuracy of UIC results and may be more representative of the population choosing a plant-based/vegan diet. A limitation to accuracy of the dietary analysis information is the date of analysis of foods in the Australian Food Composition Database (AFCD), older foods may have been analysed up to 25 years ago which will not account for more recent changes in iodine levels in foods. As the AFCD averages iodine concentration in foods from different sources such as milk and eggs, regional variations in iodine concentration are not known. The dietary analysis was based on two days of intake which may have been unusual or incomplete or may not represent the usual diet of the participants.

## 5. Conclusions

Data from the present study indicate that overall women of reproductive age consuming both an omnivorous diet and a vegan/plant-based diet may be at risk of inadequate iodine intake with women choosing a vegan/plant-based diet having lower iodine status than omnivores. Low iodine status in women of reproductive age is a potential risk to the intellectual development of future offspring. Our results indicate that a larger study is needed to determine iodine status of women in South Australia which includes women who are taking iodine containing supplements. Increasing the intake of iodine in the community may require increased food fortification and increased public awareness of iodine requirements and sources including the benefits of purchasing iodised salt over other salts. These results also suggest that fortification of other key foods such as plant-based milk-substitutes to the average level of iodine contained in dairy milk and fortification of new salts in the food supply would improve iodine status of women choosing a vegan/plant-based diet.

## Figures and Tables

**Table 1:** Characteristics of the plant based and omnivore groups.

Variable	Vegan/Plant Based Median and IQR *n* = 31	Omnivore Median and IQR *n* = 26	*p*
Age (years)	32 (23–41)	29 (21–35)	NS
Weight (Kg)	62 (57–67)	64 (58–79)	NS
BMI (kg/m^2^)	21 (20–24)	22 (21–27)	NS

NS—not significant.

**Table 2:** Urinary iodine concentration.

Iodine	Vegan/Plant Based *n* = 31	Omnivore *n* = 26	*p*
UIC (µg/L)	44 (26–66)	64 (40–88)	*p* = 0.044

Data are median and IQR

**Table 3:** Iodine containing food intake and milk alternatives.

Food	Vegan/Plant Based (*n* = 28)	Omnivore (*n* = 22)	*p*	No of Vegan/ Plant-Based Consumers	No of Omnivore Consumers
Iodised Salt				4	4
Iodised Bread Intake (g/day)	28 (0–55)	36 (10–75)	NS	17	17
Plant Milk (mL/day)	170 (5–291)	0 (0–0)	-	22	3
Dairy Milk servings/day ^	0 (0–0)	0.5 (0.3–1.2)	-	0 *	19
Total Dairy servings/day **	0 (0–0)	1.3 (0.7–1.8)	-	0 *	21
Egg (g/day)	0 (0–1)	5 (0–38)	*p* = 0.001	0 *	13
Fish servings/day (100 g raw fish = 1 serve)	0 (0–0)	0.00 (0.00–0.24)	-	1	8
Seaweed (g/day)	0 (0–0)	0 (0–0)	-	6	0

Data are median and IQR. * Only trace amounts of this food found in food records. NS = not significant. ^ Approximately 270 mL milk or 300 mg calcium based on Foodworks serving size analysis software. ** Approximately 270 mL milk, 40 g cheese, 150 g yoghurt or a serve equal to 300 mg calcium.

**Table 4:** Macronutrient intake comparisons for vegan/plant-based and omnivore groups.

Macronutrient	Vegan/Plant Based *n* = 28	Omnivore *n* = 22	*p*
Energy (kJ/day)	7146 (6095–8370)	7162 (6257–9136)	NS
Protein (g/day)	59 (51–69)	82 (67–95)	*p* = 0.001
Total Fat (g/day)	64 (53–81)	77 (68–91)	NS
Saturated fat (g/day)	16 (9–26)	28 (23–35)	*p* = 0.001
% kJ from sat fat	9.2 (±4.5) *	13.8 (±4.0) *	*p* = 0.000
Trans fat (g/day)	0.3 (0.2–0.5)	1.1 (0.8–1.4)	*p* = 0.000
VLC N3 PUFA (mg)	0 (0–110)	120 (6–400)	*p* = 0.000
ALA (g)	2.13 (1.44–2.99)	1.51 (0.97–2.36)	*p* = 0.048
Cholesterol ^^ (mg/day)	5.2 (1.1–15.7)	249 (161–365)	*p* = 0.000
Carbohydrate (g/day)	205 ± 75 *	181 ± 55 *	NS
Sugar (g/day)	75 (±31) *	72 (±40) *	NS
Alcohol (g/day)	0 (0–0.3)	0 (0–7)	NS
Fibre (g/day)	37 (29–56)	23 (20–28)	*p* = 0.000

Data are median and IQR unless otherwise specified. * Mean and standard deviation. ^^ small amounts of cholesterol were found in the vegan analysis, where participants had selected a standard food where the Foodworks recipe contained butter or eggs in the ingredients. NS—not significant. ALA—Alpha Linolenic Acid, VLC N3 PUFA—Very Long Chain Omega 3 Polyunsaturated Fatty Acids.

**Table 5:** Micronutrient intake comparisons.

Nutrients	Vegan/Plant Based *n* = 28	Omnivore *n* = 22	*p*	EAR ^	Intake RDI ^
Iodine (µg/day)	78 (62–91)	125 (86–175)	*p* = 0.000	100	150
Selenium (µg/day)	38 (32–64)	63 (51–83)	*p* = 0.005	50	60
Iron (mg/day)	14.2 (10.8–19.8)	9.8 (7.8–13.1)	*p* = 0.001	8	18
Zinc (mg/day)	8.2 (5.6–9.6)	9.8 (7.5–12.4)	*p* = 0.053	6.5	8
Calcium (mg/day)	658 (±258) *	772 (±331) *	NS	840	1000
Magnesium (mg/day)	483 (297–543)	315 (211–455)	*p* = 0.007	255–265	310–320
Sodium (mg/day)	1692 (±823) *	2439 (±932) *	*p* = 0.005	-	-
Potassium (mg/day)	3464 (2745–3966)	3296 (2256–4085)	NS	2800 AI ^	-
Thiamin (mg/day)	1.41 (1.01–1.72)	1.04 (0.87–1.39)	NS	0.9	1.1
Niacin (mg/day)	14.5 (12.2–22.2)	19.4 (13.6–24.9)	NS	11	14
B6 (mg/day)	1.52 (1.12–2.54)	1.48 (1.08–1.93)	NS	1.1	1.3
B12 (µg/day)	0.86 (0.15–1.83)	4.00 (2.41–5.35)	*p* = 0.000	2.0	2.4
Total Vit A equivalents (µg/day)	1497 (825–1907)	944 (642–1676)	NS	500	700
Riboflavin (mg/day)	1.02 (0.68–1.42)	1.15 (0.82–1.78)	NS	0.9	1.3
Vit C (mg/day)	146 (89–250)	88 (50–132)	*p* = 0.006	30	45
Vit E (mg/day)	18 (13.2–29.5)	11.5 (7–15)	*p* < 0.0001	7	-
Total Folate + Folic acid ^^ (µg/day)	508 (406–681)	423 (260–538)	*p* = 0.039	320	400
Food folate (µg/day)	411 (330–533)	258 (167–376)	*p* = 0.002	-	

Abbreviations: estimated average requirement (EAR); recommended daily intake (RDI). Data are median and IQR unless otherwise specified. * Mean ± SD. AI—adequate intake. NS—not significant. ^ NRV—Nutrient Reference Values for Australia and New Zealand (NHMRC). ^^ Folic acid contained in fortified foods such as bread.
